# Subtractive Genomics Approach for Identification of Novel Therapeutic Drug Targets in *Mycoplasma genitalium*

**DOI:** 10.3390/pathogens10080921

**Published:** 2021-07-21

**Authors:** Abiodun Joseph Fatoba, Moses Okpeku, Matthew Adekunle Adeleke

**Affiliations:** Discipline of Genetics, School of Life Sciences, University of KwaZulu-Natal, Westville, P/Bag X54001, Durban 4000, South Africa; okpekum@ukzn.ac.za

**Keywords:** *Mycoplasma genitalium*, drug targets, metabolic pathways, pathogen, proteins, subtractive genomics

## Abstract

*Mycoplasma genitalium* infection is a sexually transmitted infection that causes urethritis, cervicitis, and pelvic inflammatory disease (PID) in men and women. The global rise in antimicrobial resistance against recommended antibiotics for the treatment of *M. genitalium* infection has triggered the need to explore novel drug targets against this pathogen. The application of a bioinformatics approach through subtractive genomics has proven highly instrumental in predicting novel therapeutic targets against a pathogen. This study aimed to identify essential and non-homologous proteins with unique metabolic pathways in the pathogen that could serve as novel drug targets. Based on this, a manual comparison of the metabolic pathways of *M. genitalium* and the human host was done, generating nine pathogen-specific metabolic pathways. Additionally, the analysis of the whole proteome of *M. genitalium* using different bioinformatics databases generated 21 essential, non-homologous, and cytoplasmic proteins involved in nine pathogen-specific metabolic pathways. The further screening of these 21 cytoplasmic proteins in the DrugBank database generated 13 druggable proteins, which showed similarity with FDA-approved and experimental small-molecule drugs. A total of seven proteins that are involved in seven different pathogen-specific metabolic pathways were finally selected as novel putative drug targets after further analysis. Therefore, these proposed drug targets could aid in the design of potent drugs that may inhibit the functionality of these pathogen-specific metabolic pathways and, as such, lead to the eradication of this pathogen.

## 1. Introduction

*Mycoplasma genitalium* is an emerging cause of sexually transmitted infections (STIs) around the globe and has been implicated in urogenital infections of both men and women [1], which include urethritis [2], cervicitis [3], pelvic inflammatory disease (PID) [4], and preterm birth [5]. The pathogen is responsible for 30% of persistent non-gonococcal urethritis cases [6]. Coinfections of *M. genitalium* with other human STIs such as human immunodeficiency virus (HIV), *Chlamydia trachomatis,* and *Neisseria gonorrhoeae* have been reported in different studies [7,8,9,10]. Due to the clinical significance of *M. genitalium* infection, a guideline for its diagnosis, treatment, and management was included in the Sexually Transmitted Diseases Treatment Guidelines, 2015 by the Centers for Disease Control and Prevention (CDC) [11].

Despite these guidelines, the treatment of *M. genitalium* infection is still challenging [12]. This pathogen’s lack of cell wall has exempted the use of certain antibiotics such as the beta-lactams (penicillins and cephalosporins) that target cell wall biosynthesis [13]. The commonly used antibiotics that have proven effective in treating this infection include macrolides, tetracycline, and quinolones [14,15]. However, the rapid emergence of antimicrobial resistance against these antibiotics has become worrisome. For example, the recommended 1 g single dose of azithromycin for the treatment of non-gonococcal urethritis (GNU) has not only proven ineffective against *M. genitalium* but has also led to macrolide-resistant *M. genitalium* [16,17]. Additionally, the second-line treatment antibiotics (fluoroquinolone moxifloxacin) are effective against *M. genitalium,* but resistance against these antibiotics has also been reported in different countries [18,19,20,21]. The global rise in antimicrobial resistance among *M. genitalium* has necessitated the need to explore novel drug targets in this pathogen that can help to curtail this deadly infection.

The advances in computational biology and bioinformatics approaches through the use of omics data such as proteomics, metabolomics, and genomics have been instrumental in drug discovery as they reduce the cost and time needed for in vivo and wet-lab screening for drug design [22]. One of such bioinformatics approaches is subtractive genomics, which entails comparing pathogen and host proteomes to identify non-host proteins with unique metabolic pathways that are critical to the survival of the pathogens. These essential proteins, which must show no cross-reaction with the host, have been proposed as potential drug targets. This approach has been used in different studies to identify potential drug targets and vaccine candidates against different pathogenic bacteria [23,24,25]. A previous study by Butt et al. [26] used this same approach to predict two unique metabolic pathways (bacteria secretion system and phosphotransferase system (PTS)) of *M. genitalium* as potential drug targets. However, the metabolic pathways of *M. genitalium* in the KEGG database have been updated since then and now have 53 metabolic pathways compared to the 43 reported by Butt et al. [26]. Therefore, this demands further study to explore additional potential drug targets that could help in drug design against this pathogen.

In this study, we explored the identification of potential drug targets from the whole proteome of *M. genitalium*. The proteome of the pathogen was mined using a subtractive genomics approach. Cytoplasmic proteins with unique metabolic pathways that play critical roles in the pathogen’s survival with no potential of cross-reaction in the host were selected as novel drug targets. These proteins, when further experimentally confirmed, could aid in drug development against *M. genitalium* infection.

## 2. Results and Discussion

### 2.1. Sequence Retrieval, Filtering and Identification of Essential Proteins

The 511 complete protein sequences of *M. genitalium* str. G37 (Supplementary File S2) retrieved from UniProtKB were reduced to 488 sequences after removing redundant sequences by CD-HIT at a 90% threshold. The 488 sequences were further subjected to Geptop server, which predicted 381 sequences as essential proteins (Supplementary File S3). Essential proteins are mostly present in antimicrobial compounds and are promising targets for drug design [27].

### 2.2. Identification of Essential and Non-Homologous Proteins

**Figure 1:**
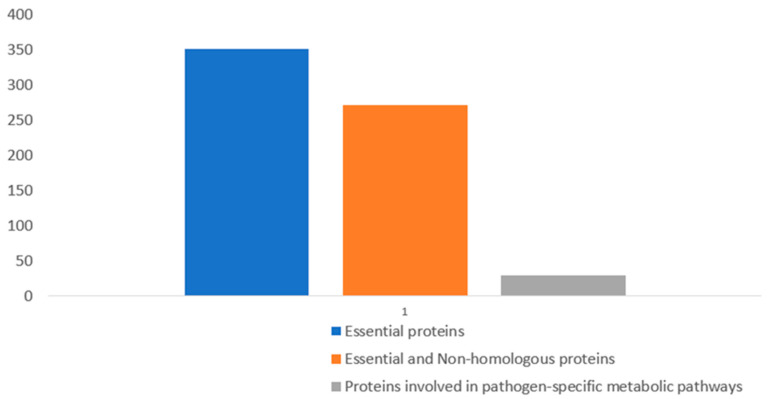
Result showing the gradual reduction of essential proteins after BLASTp analysis.

Essential proteins that contribute to the survival of a pathogen while in the host body are potential drug targets, but such proteins should be non-homologous to host (human) proteins to prevent the likelihood of adverse drug effects [28,29]. Based on this, the 381 essential proteins were subjected to BLASTp against human (*Homo sapiens*) proteins, and this generated 272 sequences that are regarded as essential and non-homologous proteins (Figure 1). The sequences of these proteins can be found in Supplementary File S4.

### 2.3. Metabolic Pathways Analysis

**Figure 2:**
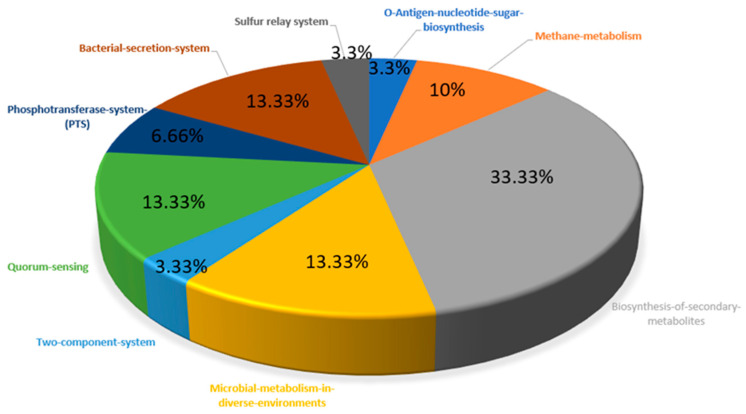
Distribution of essential and non-homologous proteins involved in the unique metabolic pathways of *M. genitalium*.

The KEGG database contains 53 and 343 metabolic pathways for *M. genitalium* and *H. sapiens*, respectively. The manual comparison of the metabolic pathways of the pathogen and host showed nine metabolic pathways to be unique to the pathogen, and as such, they were regarded as pathogen-specific metabolic pathways. The remaining 44 metabolic pathways were common to both the pathogen and the host. Out of the 272 essential and non-homologous proteins of *M. genitalium*, 30 were predicted via BLASTp in the KASS server to be involved in the nine pathogen-specific metabolic pathways (Table 1). The distribution of unique metabolic pathways among the essential and non-homologous proteins is also shown in Figure 2.

### 2.4. Prediction of Subcellular Localization

Subcellular localization provides information and insight about the location and function of an essential protein. Cytoplasmic proteins are more suitable as drug targets [29]. Out of the 30 essential and non-homologous proteins involved in unique metabolic pathways, 21 proteins were predicted by CELLO and PSLpred as cytoplasmic proteins, as shown in Supplementary Table S1.

### 2.5. Screening for Druggable Proteins and Detection of Novel Drug Targets

Druggability shows the likelihood of a small-molecule drug to modulate the activity of a therapeutic target [30]. Subjecting the 21 cytoplasmic proteins to BLASTp against druggable proteins sequences in DrugBank Database generated 13 druggable *M. genitalium* proteins that shared similarity with FDA-approved and experimental small-molecule drugs (Table 2). All these 13 druggable proteins can serve as drug targets in the design of antibiotics drug against this pathogen. Moreover, the remaining eight proteins (seven characterised proteins and one uncharacterised protein) that showed no similarity with any known drug/drug targets in the DrugBank database were termed novel drug targets (Table 3), and the characterised proteins were selected for further analysis. A similar approach has been used by different authors to detect novel drug targets against different pathogens [23,24,31]. The unique metabolic pathways of these proposed novel drug targets include the following: microbial metabolism in diverse environments, two-component system, biosynthesis of secondary metabolites, O-antigen nucleotide sugar biosynthesis, quorum sensing, bacterial secretion, sulphur relay system, and methane metabolism (Table 3). Brief information on some of the unique metabolic pathways as proposed novel drug targets in bacteria is summarised below.

#### 2.5.1. Methane Metabolism

Energy production in the form of ATP is essential for maintaining cellular activities in all living organisms. Methane metabolism, which entails the breaking down of one-carbon compound (methane) to obtain energy, is one of the unique metabolic pathways in bacteria. One of the proteins predicted in this study participates in the methane metabolism pathway. This unique pathway is crucial to the survival of bacteria and has been suggested as a potential drug target in different bacteria such as *Mycobacterium tuberculosis*, *Mycobacterium avium*, and *Enterococcus faecium* [32,33,34].

#### 2.5.2. Biosynthesis of Secondary Metabolites

Secondary metabolites are small bioactive molecules produced in bacteria during their stationary phase of growth [35]. They are not essentially important for the growth and survival of bacteria, but they provide competitive advantages [36]. Three proteins from this study predicted to be involved in the biosynthesis of secondary metabolites are unique to *M. genitalium*. This is similar to reports from other studies where the proteins associated with this pathway have been described to be essential, non-homologous, and unique to different bacteria pathogens such as *Vibrio parahaemolyticus* and *Staphylococcus saprophyticus* [23,37]. Different studies have reported the manipulation of bacterial secondary metabolites pathways and their use as antibiotics, antiviral drugs, and other pharmaceutical drugs [38,39,40,41].

#### 2.5.3. Quorum Sensing

Bacteria use quorum-sensing cell communication to regulate several cellular processes such as virulence, biofilm formation, pathogenicity, and drug resistance [42,43,44]. Other activities controlled by quorum sensing include antibiotic production and sporulation [45]. This present study showed that the protein translocase subunit that was predicted as a novel drug target is involved in the quorum-sensing metabolic pathway in *M. genitalium*. A recent study by Daubenspeck et al. [46] indicated that biofilm formation contributes to antibiotics resistance in *M. genitalium*. Due to the significant role of quorum sensing in biofilm formation in most bacteria, proteins from this metabolic pathway have been proposed as potential therapeutic targets [47,48].

#### 2.5.4. Two-Component System (TCS)

Bacteria widely use two-component system (TCS) to respond to environmental signals [49]. TCS consists of histidine kinase and a sensor regulator. It is involved in the expression of virulence and antibiotic-resistance responses in pathogenic bacteria [49]. For example, VicRK (a TCS) has been reported to regulate biofilm formation, virulence, and lipid metabolism [50,51]. Similarly, the VanS/VanR two-component system controls the resistance of enterococci to vancomycin [52]. Though one of the seven proteins predicted as novel drug targets in this study is involved in the TCS metabolic pathway, no TCS has been reported so far in *M. genitalium* in the literature [53]. However, serine/threonine kinases (STK) and phosphatases (STP), which are signal transduction systems, have been indicated to perform similar functions as a TCS [54]. A report by Martinez et al. [53] also showed that STP is involved in the virulence of *M. genitalium*. Other TCSs such as DegS-DegU, Pho regulon (such as PhoR-PhoP, PhoR–PhoS, and PhoP-PhoQ), and QseC-QsecB—which are present in *Bacillus subtilis* [55,56], *Corynebacterium glutamicum* [57], *Shigella* species [58], and enterohaemorrhagic *E. coli* [59]—are involved in the pathogenesis and biofilm formation of bacteria.

As such, a TCS serves as a potential antibiotic target [60].

#### 2.5.5. Bacterial Secretion System

The bacterial secretion system facilitates the transport of proteins from the cytoplasm of most bacteria either to their host environment or directly into their host cells. They aid in bacteria’s virulence and pathogenicity, leading to severe damage to the host cell [61]. In this study, the protein translocase subunit was found to be involved in the bacterial secretion system metabolic pathway, which was similar to a previous study by Butt et al. [26] that also predicted the bacteria secretion system as a novel drug target to be considered in the design of a potent antimicrobial agent against *M. genitalium*. Different classes of this secretion system in bacteria (type I–VII) have been regarded as potential drug targets [62,63,64].

### 2.6. Anti-Target Analysis of Novel Drug Targets and 3D Structure Analysis

Toxicity and other adverse drug reactions could cause drug withdrawal from public use [65,66]. To ensure the effectiveness and safety of potential drugs, it is essential to screen the proposed drug targets against possible anti-target proteins. The shortlisted eight novel drug target proteins showed no similarity with the 210 human anti-target proteins using NCBI BLASTp (Table 3). The novelty of these proposed novel drug targets was also confirmed by their low query coverage and/or percentage identity with known 3D protein structures in the PDB (Supplementary File S5). In summary, seven essential, non-homologous, and cytoplasmic proteins that are involved in seven unique pathways (Table 3) were proposed as potential drug targets from this study. Our study correlates with previous studies where some of these proposed unique metabolic pathways have been regarded as drug targets in different bacteria [67,68,69].

## 3. Materials and Methods

**Figure 3:**
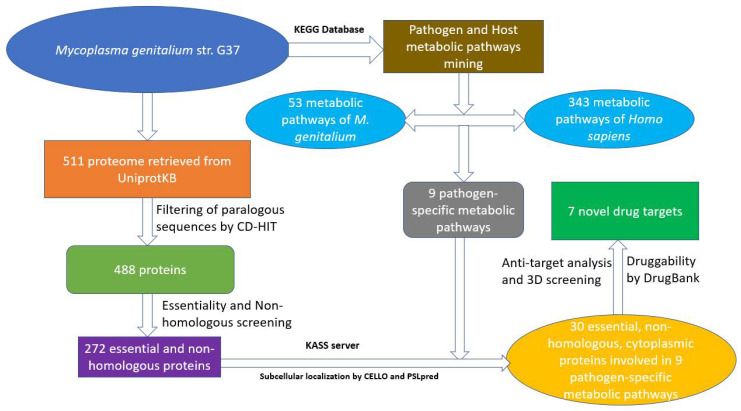
The workflow showing the prediction of novel drug targets.

The subtractive genomics approach is a reliable method of prioritising potential drug targets from the whole proteome of a pathogen. A flow-chart showing all the steps carried out in this work is shown in Figure 3.

### 3.1. Sequence Retrieval and Removal of Paralogous Sequences

The whole proteome of *Mycoplasma genitalium* str. G37 consisting of 511 protein sequences was downloaded from the UniProtKB database (https://www.uniprot.org/, accessed on 15 April 2021). Out of these, paralogous sequences with a cut-off score of 0.9 (90% identity) were excluded from the protein sequences using the CD-HIT server. CD-HIT is a server that clusters biological sequences, thereby reducing the redundancy of protein sequences [70].

### 3.2. Prediction of Essential Proteins

After excluding paralogous sequences, the remaining protein sequences were subjected to Geptop 2.0 server to screen for essential proteins, which play a critical role in the survival of a pathogen. With an essentiality cut-off score of 0.24, the Geptop server identifies essential genes in bacteria by comparing query protein ortholog and phylogeny with experimentally determined essential gene datasets present in the Database of Essential Genes (DEG) [71].

### 3.3. Screening for Non-Homologous Protein

Using the default parameters of NCBI BLASTp [72], the predicted essential proteins were screened against the human protein (*Homo sapiens*). Essential proteins showing no hits and those with an identity of <20% but a query coverage of >70% were selected as non-homologous and used for further analysis.

### 3.4. Analysis of Metabolic Pathways

The Kyoto Encyclopedia of Genes and Genome (KEGG) database [73], which contains the complete metabolic pathways of most living organisms, was used to retrieve the metabolic pathways of *M. genitalium str.* G37 and human host (*Homo sapiens*) using the KEGG three letter organism codes ‘mge’ and ‘hsa,’ respectively. The metabolic pathways of the pathogen and host were compared, and those pathways found in the pathogen but absent in the human host were regarded as unique pathways. Common pathways are those found in both a pathogen and its human host. The essential and non-homologous proteins of *M. genitalium* were subjected to BLASTp present in KEGG Automatic Annotation Server (KASS), an automatic genome annotation and pathway reconstruction too [74]. KASS provides KEGG Orthology identifiers (KO) and information about the metabolic pathways. The essential and non-homologous proteins of *M. genitalium* with unique metabolic pathways were selected for subsequent analysis.

### 3.5. Prediction of Subcellular Localization

Sub-cellular localisation prediction classifies proteins into different positions, including cytoplasm, inner membrane, periplasmic, and outer membrane. Proteins located in the cytoplasm and outer membrane are potential drug and vaccine targets, respectively. CELLO and PSLpred servers that employ support vector machine (SVM) and hybrid SVM-based approaches with prediction accuracies of 89% and 91%, respectively, were used to predict subcellular localisation [75,76].

### 3.6. Druggability Analysis and Detection of Novel Drug Targets

To identify druggable proteins, the essential and non-homologous proteins of *M. genitalium* with unique metabolic pathways were screened against the DrugBank database using the default parameters. The DrugBank database consists of 14,542 drug entries, and these include FDA-approved drugs, nutraceuticals, and experimental drugs [77]. Novel putative drug targets that showed no hits with known drug/drug targets in the DrugBank were selected for further analysis.

### 3.7. Anti-Target Analysis of Novel Drug Targets and 3D Structure Analysis

Anti-target proteins are important proteins in the host cell that could dock with potential therapeutic compounds designed against a pathogen. In humans, 210 of such anti-target proteins (Supplementary File S1) have been reported in the literature, including P-glycoprotein (P-gly), adrenergic receptor, dopaminergic receptor, and ether-a-go-go-related protein. To avoid the cross-reaction of these anti-target proteins with the proposed drug targets, which could lead to toxic effects, the novel drug targets were subjected to NCBI BLASTp against these 210 anti-targets proteins with the following parameters: an E-value of <0.005, a query coverage of >30%, and an identity of <25%. To further verify the novelty of these novel drug targets, their sequences were screened against known 3D structures in the Protein Data Bank (PDB) using PSI-BLAST in NCBI.

## 4. Conclusions

The rapid emergence of antimicrobial resistance among Gram-positive bacteria has triggered the need to explore novel drug targets that could assist in designing new antimicrobial agents. This present study provided seven cytoplasmic proteins of *Mycoplasma genitalium* as novel putative drug targets. These proteins are involved in pathogen-specific metabolic pathways, some of which have been reported as therapeutic targets in different microorganisms. The lack of similarities of these proposed novel putative drug targets with human proteome and anti-targets has further reduced the chances of interaction between drugs and human proteins. Therefore, the design of new antimicrobial agents that would inhibit these novel putative drug targets could provide effective control against *M. genitalium* infection, though these putative drug targets still need to be subjected to further analysis and be experimentally validated.

## Figures and Tables

**Table 1:** List of unique metabolic pathways in *Mycoplasma genitalium* with their KEGG Ortholog.

Protein ID	Protein Name	KO Assignment	Unique Metabolic Pathways
P22746	Bifunctional oligoribonuclease and PAP phosphatase	K06881	mge01120-Microbial metabolism in diverse environments
P35888	Chromosomal replication initiator protein	K02313	mge02020-Two component system
P47259	Bifunctional protein FolD	K01491	mge01120-Microbial-metabolism-in-diverse-environments
P47269	Fructose-bisphosphate aldolase	K01624	mge00680-Methane-metabolism, mge01110-Biosynthesis-of-secondary-metabolites, mge01120-Microbial-metabolism-in-diverse-environments
P47287	Phosphocarrier protein	K02784	mge02060-Phosphotransferase-system-(PTS)
P47299	Phosphomannomutase	K01840	mge00541-O-Antigen-nucleotide-sugar-biosynthesis, mge01110-Biosynthesis-of-secondary-metabolites
P47301	Uncharacterised protein	K03073	mge02024-Quorum-sensing, mge03070-Bacterial-secretion-system
P47315	PTS system	K20118	mge02060-Phosphotransferase-system-(PTS)
P47318	Protein translocase subunit	K03070	mge02024-Quorum-sensing, mge03070-Bacterial-secretion-system
P47323	Oligopeptide transport system permease protein	K15581	mge02024-Quorum-sensing
P47324	Oligopeptide transport system permease protein	K15582	mge02024-Quorum-sensing
P47357	Glucose-6-phosphate isomerase	K01810	mge01110-Biosynthesis-of-secondary-metabolites, mge01120-Microbial-metabolism-in-diverse-environments
P47391	Bifunctional riboflavin kinase	K11753	mge01110-Biosynthesis-of-secondary-metabolites
P47416	Protein translocase subunit	K03076	mge02024-Quorum-sensing, mge03070-Bacterial-secretion-system
P47489	Glycerol-3-phosphate acyltransferase	K08591	mge01110-Biosynthesis-of-secondary-metabolites
P47514	Dihydrolipoyllysine-residue acetyltransferase	K00627	mge01110-Biosynthesis-of-secondary-metabolites, mge01120-Microbial-metabolism-in-diverse-environments
P47515	Pyruvate dehydrogenase E1 component subunit beta	K00162	mge01110-Biosynthesis-of-secondary-metabolites, mge01120-Microbial-metabolism-in-diverse-environments
P47516	Pyruvate dehydrogenase E1 component subunit alpha	K00161	mge01110-Biosynthesis-of-secondary-metabolites, mge01120-Microbial-metabolism-in-diverse-environments
P47529	Acyl carrier protein homolog	K02078	mge01110-Biosynthesis-of-secondary-metabolites
P47541	Phosphate acetyltransferase	K00625	mge00680-Methane-metabolism mge01120-Microbial-metabolism-in-diverse-environments
P47599	Acetate kinase	K00925	mge00680-Methane-metabolism, mge01120-Microbial-metabolism-in-diverse-environments
P47612	Probable tRNA sulfurtransferase	K03151	mge04122-Sulfur relay system
P47636	Probable ribose-5-phosphate isomerase B	K01808	mge01110-Biosynthesis-of-secondary-metabolites, mge01120-Microbial-metabolism-in-diverse-environments
P47668	Phosphoenolpyruvate-protein phosphotransferase	K08483	mge02060-Phosphotransferase-system-(PTS)
P47669	2,3-bisphosphoglycerate-independent phosphoglycerate mutase	K15633	mge00680-Methane-metabolism, mge01110-Biosynthesis-of-secondary-metabolites, mge01120-Microbial-metabolism-in-diverse-environments
P47696	Hypoxanthine-guanine phosphoribosyltransferase	K00760	mge01110-Biosynthesis-of-secondary-metabolites
P47702	Membrane protein insertase	K03217	mge02024-Quorum-sensing, mge03070-Bacterial-secretion-system
P58061	Probable protein-export membrane protein	K03075	mge02024-Quorum-sensing, mge03070-Bacterial-secretion-system
Q49409	Uncharacterised protein	K12257	mge02024-Quorum-sensing, mge03070-Bacterial-secretion-system
Q49427	Phosphate acyltransferase	K03621	mge01110-Biosynthesis-of-secondary-metabolites

**Table 2:** List of 13 druggable proteins of *Mycoplasma genitalium*.

Protein ID	Drug Name	DrugBank ID	Drug Group	Subcellular Localisation
P47259	Tetrahydrofolic acid	DB00116	Nutraceutical	Cytoplasmic
	NADH	DB00157	Approved	
	LY374571	DB02358	Experimental	
	Nicotinamide adenine dinucleotide phosphate	DB03461	Experimental	
	LY249543	DB04322	Experimental	
P47269	Phosphoglycolohydroxamic acid	DB03026	Experimental	Cytoplasmic
P47287	Dexfosfoserine	DB04522	Experimental	Cytoplasmic
P47357	N-Bromoacetyl-aminoethyl Phosphate	DB02257	Experimental	Cytoplasmic
	5-Phosphoarabinonic acid	DB03042	Experimental	
P47514	NADH	DB00157	Approved	Cytoplasmic
	Radicicol	DB03758	Experimental	
	Dihydrolipoic acid	DB03760	Experimental	
P47515	Pyruvic acid	DB00119	Approved	Cytoplasmic
	NADH	DB00157	Approved	
P47516	NADH	DB00157	Approved	Cytoplasmic
P47541	Acetylphosphate	DB02897	Experimental	Cytoplasmic
P47599	Formic acid	DB01942	Experimental	Cytoplasmic
	Adenosine-5′-[Beta, Gamma-Methylene]Triphosphate	DB03909	Experimental	
P47636	L-cysteic acid	DB03661	Experimental	Cytoplasmic
	4-phospho-D-erythronic acid	DB03108	Experimental	
	4-Phospho-D-erythronohydroxamic acid	DB04496	Experimental	
P47668	Diethylene glycol diethyl ether	DB08357	Experimental	Cytoplasmic
P47669	2-phospho-D-glyceric acid	DB01709	Experimental	Cytoplasmic
	3-phospho-D-glyceric acid	DB04510	Experimental	
P47696	Inosinic acid	DB04566	Experimental	Cytoplasmic
	5-O-phosphono-alpha-D-ribofuranosyl diphosphate	DB01632	Approved	
	Formycin B	DB04198	Experimental	
	Mercaptopurine	DB01033	Approved	
	Tioguanine	DB00352	Approved	
	Azathioprine	DB00993	Approved	
	Artenimol	DB11638	Approved	

**Table 3:** List of novel drug targets in *Mycoplasma genitalium* showing no similarity with human anti-target proteins.

Protein ID	Protein Name	Subcellular Localisation	Unique Metabolic Pathways	Anti-Target Similarity
P22746	Bifunctional oligoribonuclease and PAP phosphatase	Cytoplasmic	mge01120-Microbial metabolism in diverse environments	No
P35888	Chromosomal replication initiator protein	Cytoplasmic	mge02020-Two component system	No
P47299	Phosphomannomutase	Cytoplasmic	mge01110-Biosynthesis of secondary metabolites	No
			mge00541-O-Antigen nucleotide sugar biosynthesis	
P47318	Protein translocase subunit	Cytoplasmic	mge02024-Quorum-sensing	No
			mge03070-Bacterial secretion system	
P47529	Acyl carrier protein homolog	Cytoplasmic	mge01110-Biosynthesis of secondary metabolites	No
P47612	Probable tRNA sulfurtransferase	Cytoplasmic	mge04122-Sulfur relay system	No
Q49427	Phosphate acyltransferase	Cytoplasmic	mge01110-Biosynthesis of secondary metabolites	No

## Data Availability

Not applicable.

## Supplementary Materials

The following are available online at https://www.mdpi.com/article/10.3390/pathogens10080921/s1, Table S1: List of consensus cytoplasmic proteins generated by CELLO and PSLpred servers; File S1: List of anti-target protein sequences in human, File S2: The complete protein sequences of *M. genitalium* str. G37, File S3: List of essential proteins predicted by Geptop server, File S4: List of essential and non-homologous protein, File S5: List of known 3D protein structure generated by PSI-BLAST.
