# Supplementary material for: Subtractive Genomics Approach for Identification of Novel Therapeutic Drug Targets in Mycoplasma genitalium
Source: Pathogens. 2021 Jul 21;10(8):921. doi: 10.3390/pathogens10080921 (PMC8402164; doi:10.3390/pathogens10080921)
Supplement: Supplementary file 1 [file pathogens-10-00921-s001.zip › Table S1.pdf]

Table S1. List of consensus cytoplasmic proteins by CELLO and PSLpred servers.

Protein ID	PSLpred	CELLO
P22746	Cytoplasmic	Cytoplasmic
P35888	Cytoplasmic	Cytoplasmic
P47259	Cytoplasmic	Cytoplasmic
P47269	Cytoplasmic	Cytoplasmic
P47287	Cytoplasmic	Cytoplasmic
P47299	Cytoplasmic	Cytoplasmic
P47301	Cytoplasmic	Cytoplasmic
P47315	Inner Membrane	Inner membrane
P47318	Cytoplasmic	Cytoplasmic
P47323	Cytoplasmic	Inner membrane
P47324	Cytoplasmic	Inner membrane
P47357	Cytoplasmic	Cytoplasmic
P47391	Outer Membrane	Outer membrane
P47416	Inner Membrane	Inner membrane
P47489	Inner Membrane	Inner membrane
P47514	Cytoplasmic	Cytoplasmic
P47515	Cytoplasmic	Cytoplasmic
P47516	Cytoplasmic	Cytoplasmic
P47529	Cytoplasmic	Cytoplasmic
P47541	Cytoplasmic	Cytoplasmic
P47599	Cytoplasmic	Cytoplasmic
P47612	Cytoplasmic	Cytoplasmic
P47636	Cytoplasmic	Cytoplasmic
P47668	Cytoplasmic	Cytoplasmic
P47669	Cytoplasmic	Cytoplasmic
P47696	Cytoplasmic	Cytoplasmic
P47702	Cytoplasmic	Inner membrane
P58061	Cytoplasmic	Inner membrane
Q49409	Inner Membrane	Inner membrane
Q49427	Cytoplasmic	Cytoplasmic
